# Protocolized polyoma BK viral load monitoring and high-dose immunoglobulin treatment in children after kidney transplant

**DOI:** 10.1093/ckj/sfad293

**Published:** 2023-11-27

**Authors:** Shirley Pollack, Moran Plonsky-Toder, Rami Tibi, Renata Yakubov, Irina Libinson-Zebegret, Daniella Magen

**Affiliations:** Pediatric Nephrology Institute, Ruth Children's Hospital, Rambam Health Care Campus, Technion Faculty of Medicine, Haifa, Israel; Pediatric Nephrology Institute, Ruth Children's Hospital, Rambam Health Care Campus, Technion Faculty of Medicine, Haifa, Israel; Pediatric Nephrology Institute, Ruth Children's Hospital, Rambam Health Care Campus, Technion Faculty of Medicine, Haifa, Israel; Pediatric Nephrology Institute, Ruth Children's Hospital, Rambam Health Care Campus, Technion Faculty of Medicine, Haifa, Israel; Pediatric Nephrology Institute, Ruth Children's Hospital, Rambam Health Care Campus, Technion Faculty of Medicine, Haifa, Israel; Pediatric Nephrology Institute, Ruth Children's Hospital, Rambam Health Care Campus, Technion Faculty of Medicine, Haifa, Israel

**Keywords:** BKPyVANGraft survival, IVIG, pediatric renal transplant, rejection

## Abstract

**Background:**

BKPyV virus nephropathy (BKPyVAN) is diagnosed in 5%–16% of pediatric renal transplant recipients (PRTR) and preceded by BKPyV-viruria and DNAemia. Despite the risk of irreversible transplant damage associated with BKPyVAN, evidence-based consensus guidelines for BKPyVAN prevention are still lacking. In this retrospective study, we examined the safety and efficacy of high-dose intravenous immunoglobulin (HD-IVIG) therapy for prevention of BKPyVAN in PRTR with significant BKPyV-viruria/DNAemia.

**Methods:**

Between January 2013 and December 2022, all PRTR under our care underwent routine urine and blood testing for BKPyV viral load, using specific polymerase chain reaction (PCR). BKPyV DNAemia, with <10^3^ copies/mL, with BKPyV viruria <10^7^ copies/mL, with no evidence of BKPyVAN, were managed with 50% dose reduction of mycophenolate mofetil (MMF). Patients showing no decline in BKPyV viral load within two months of MMF dose reduction were managed with HD-IVIG (2 g/kg).

**Results:**

Seventy patients were recruited during a ten-year period and 31/70 patients (44%) demonstrated significant post-transplantation BKPyV-viruria/DNAemia, while 13/31 (42%) patients were unresponsive to MMF dose reduction, and were administered HD-IVIG. Of these, 12/13 (92%) patients achieved BKPyV viral clearance within six months from completion of HD-IVIG therapy and 1/13 patient (8%) was unresponsive to HD-IVIG therapy, showing increased BKPyV viral load. There were no major adverse events associated with HD-IVIG, and none of our patients developed BKPyVAN during the study period.

**Conclusions:**

Prophylactic HD-IVIG therapy in PRTR with significant BKPyV-viruria/DNAemia unresponsive to MMF dose reduction is safe and might be effective in preventing BKPyVAN. Our findings remain to be established by large-scale prospective studies.

KEY LEARNING POINTS **What was known:**

- Primary BKPyV infection mainly occurs during childhood, and is mostly asymptomatic or manifested as a mild upper respiratory tract infection in immunocompetent hosts.

**This study adds:**

- BKPyV infection in adult renal transplant recipients mainly reflects reactivation of latent BKPyV in the patient's own urinary system, while pediatric renal transplant recipients usually encounter primary BKPyV infections through transmission from the donor kidney.
- Reduction of immunosuppressive medication is the first-line treatment for prevention of BKPyVAN with a high viral load or BKPyVAN; nevertheless, standardized guidelines are still lacking.

**Potential impact:**

- This approach might jeopardize the graft with rejection.
- HD-IVIG therapy is safe and with MMF dose reduction was found to be effective in prevention of irreversible kidney damage in a cohort of pediatric renal transplant recipients.

## INTRODUCTION

BKPyV is a non-enveloped, double-stranded DNA virus of the polyomaviruses family, named after the initials (BK) of a renal transplant recipient from whom it was first isolated. BKPyV exhibit special renal and uroepithelial tropism, where it remains latent after initial infection. While healthy individuals are generally asymptomatic during BKPyV infection, immunocompromised host, including bone marrow and renal transplant recipients, are at increased risk for accelerated viral replication, causing symptomatic and invasive disease [1].

In immunocompetent hosts, primary BKPyV infection mainly occurs during childhood, through exposure to infected respiratory or urine secretions, and is mostly asymptomatic or manifested as a mild upper respiratory tract infection. Subsequent viral spread from the respiratory tract via transient viremia may lead to BKPyV latency in renal and uroepithelial tissue. Serologic evidence for prior BKPyV infection can be found in 90% of the adult population.

The pathogenesis of BKPyV infection in immunocompromised renal transplant recipients is age-dependent. BKPyV infection in adult renal transplant recipients mainly reflects reactivation of latent BKPyV in the patient's own urinary system, while pediatric renal transplant recipients usually encounter primary BKPyV infections through transmission from the donor kidney. Regardless of age, BKPyV infection or reactivation in renal transplant recipients results in enhanced viral replication within the graft tissue, followed by massive urinary viral shedding. BKPyV-viruria precedes DNAemia by a reported lag time of 1–3 months. Significant and persistent BKPyV DNAemia may lead to overt BKPyV nephropathy (BKPyVAN) [2].

Although uncommon, BKPyVAN is a severe complication of BKPyV infection in renal transplant recipients. Although 30%–40% of adult renal transplant recipients develop post-transplantation BKPyV-viruria, followed by BKPyV DNAemia, and 10%–30% develop BKPyV DNAemia, overt BKPyVAN occurs in only 5%–8% of renal transplant recipients. Nevertheless, 80%–90% of patients with proven BKPyVAN are at risk of developing irreversible graft damage that may lead to graft loss within five years from diagnosis [2, 3]. Hence, meticulous monitoring of BKPyV viral load in patients at high risk for developing BKPyVAN is crucial.

Because most pediatric renal transplant recipients are BKPyV-naive prior to transplantation, they are at high risk for primary BKPyV infections. Approximately 50% of pediatric renal transplant recipients develop BKPyV-viruria/DNAemia during the first year post-transplantation [4]. Nevertheless, epidemiologic data regarding the risk of BKPyVAN in children compared to adults is scarce, and there are no standardized guidelines for diagnosis, prevention, and treatment of BKPyV infections in pediatric renal transplant recipients [4, 5].

Overall, identified risk factors for reactivation of latent BKPyV in renal transplant recipients include high-dose immunosuppressive medications (especially during the first post-transplantation period), tacrolimus-based immunosuppressive regimen, surgical placement of indwelling ureteral double-J stents, male sex, pediatric age, deceased donor, congenital anomalies of the urinary tract, and post-transplantation delayed graft function [4, 6–8].

The clinical diagnosis of BKPyVAN in renal transplant patients is challenging, because its most common manifestation is asymptomatic decline in renal function. Hence, confirmation by renal biopsy is useful [9, 10]. Histologic manifestations of BKPyVAN include tubulointerstitial lymphocytic infiltration resembling acute cell-mediated rejection, and basophilic nuclear viral inclusion bodies within epithelial cells. Immunohistochemistry is typically positive for Simian virus 40 large T-antigen (SV-40). The extent of tubular inflammation and interstitial atrophy is positively correlated with the risk of graft loss [3].

Preemptive monitoring of BKPyV viral load by real-time polymerase chain reaction (RT-PCR) is essential for early diagnosis of BKPyV replication. Persistently elevated BKPyV viral loads exceeding 10^7^ copies/mL in urine or 10^4^ copies/mL in blood over a three-week period are highly associated with BKPyVAN risk [11]. Of note, while blood BKPyV monitoring is considered as routine practice by most transplantation centers, the significance of urine BKPyV monitoring is controversial.

Although reduction of immunosuppressive medication is the first-line treatment for prevention of BKPyVAN in renal transplant recipients with a high viral load or BKPyVAN, standardized guidelines are still lacking [5]. Numerous reported protocols for reduction of immunosuppression include, among others, tacrolimus to cyclosporine switch, mycophenolate mofetil (MMF) dose reduction, or calcineurin inhibitors to mTOR inhibitors switch. Regardless of the chosen protocol, immunosuppressive dose reduction harbors a risk of graft rejection, thereby necessitating close surveillance of renal function [12]. Adjunct therapies of limited efficacy in renal transplant recipients with elevated BKPyV viral loads include leflunomide, an anti-inflammatory agent with anti-viral and immunosuppressive properties [13], and quinolone antibiotics [14]. Cidofovir, a nucleoside analog active against cytomegalovirus (CMV) is also effective for BKPyV infection. However, given the nephrotoxicity of cidofovir, it should be used cautiously, and limited to patients who are unresponsive to first-line treatment modalities [15, 16].

Immunoglobulins, commercially extracted from numerous blood donors, naturally contain a mixture of neutralizing antibodies against various BKPyV strains [1]. Evidence from case reports, small case series, and uncontrolled clinical studies suggest that high-dose intravenous immune globulins (HD-IVIG) effectively reduces BKPyV viral loads in adult patients who are unresponsive to immunosuppressive dose reduction [17–19]. In view of the fact that a high proportion of pediatric renal transplant recipients are BKPyV-naive prior to transplantation [4], the efficacy of HD-IVIG treatment in reducing BKPyV viral loads in children warrants further examination.

### Aims

Our study aims to determine the demographic and clinical characteristics of a cohort of pediatric renal transplant recipients followed at a tertiary referral center in northern Israel, with significant BKPyV viruria/DNAemia, to identify risk factors for enhanced BKPyV replication, and to examine the safety and efficacy of HD-IVIG therapy for BKPyV viral load and prevention of BKPyVAN.

## MATERIALS AND METHODS

In this retrospective study, all pediatric renal transplant recipients aged 20 years and younger at the time of transplantation who were followed at a Pediatric Nephrology Institute of a tertiary referral center in the north of Israel between January 2013 and December 2022 were included. The study protocol was approved by the institutional review board. Demographic, clinical, and laboratory data were retrieved from the institutional electronic medical records. Surgical protocol included double-J stenting of the transplanted ureter for the first 3–6 weeks post-transplantation. Immunosuppressive protocol included induction therapy with high-dose glucocorticoids, basiliximab, or thymoglobulin, followed by maintenance triple therapy with glucocorticoids (with dose reduction during the first 3 months post-transplantation to a dose of 5 mg/kg every other day), MMF (dose during the first two weeks of 600 mg/m^2^ × 2/d, and then after reduced to 300 mg/m^2^ × 2/d) and tacrolimus (trough levels 5–8 ng/ml). BKPyV-PCR monitoring in urine and blood was performed immediately after surgery, biweekly for the following three months, and every 1–3 months thereafter, according to clinical considerations. Isolated BKPyV-viruria up to 10^7^ copies/mL without BKPyV DNAemia was closely monitored with no intervention. BK-viruria of up to 10^7^ copies/mL with BKPyV DNAemia of up to 10^3^ copies/mL was managed MMF dose-reduction by 50%, combined with quinolone antibiotics. We chose to reduce MMF dose, in view of the reported increased risk of graft rejection associated with tacrolimus dose reduction [20]. Patients with no reduction of BK-viruria by at least two orders of magnitude and/or with persistent BKPyV DNAemia, two months after MMF dose reduction and quinolone administration, were given HD-IVIG, using a total dose of 2 g/kg, equally divided during 2–5 consecutive days. MMF dose was raised to full dose after reduction of BKPyV by at least two orders of magnitude, quinolone was stopped for all patients after two months of administration.

Statistical analysis of results was performed using the Wilcoxon rank-sum test and the Z-test.

## RESULTS

The study included 70 pediatric renal transplant recipients over a 10-year follow-up period. Patients’ age at the time of transplantation was 2–20 years (median 13 years). Cadaveric donor transplantation was performed in 40 patients, while 30 received living organ donation.

BKPyV-PCR blood and urine tests were persistently negative throughout the study period in 39 patients (56%), while 31 patients (44%) showed positive urine BKPyV-PCR during follow-up. All positive BKPyV-PCR tests were identified during the first year after transplantation, with a median time of 5 months post transplantation. Six of the BKPyV-positive patients (19%) showed isolated BKPyV-viruria of up to 10^5^ copies/mL, which cleared spontaneously within three months from diagnosis.

A total of 25/31 (81%) patients with positive urinary BKPyV-PCR suffered from increasing viral loads ranging from 1.5 × 10^6^ to 1.8 × 10^7^ copies/mL, and seven of them developed BKPyV DNAemiawith viral loads ranging from 10^4^–7.8 × 10^4^ copies/mL. All 25 patients were managed with MMF dose reduction and quinolone administration. Of these, 12 patients (48%) showed complete clearance of DNAemia and/or significant reduction of viruria, without further change of treatment protocol. One of these 25 patients (4%) developed biopsy proven acute cellular rejection after MMF dose reduction with BKPyV viral clearance. Rejection resolved with appropriate immunosuppressive treatment and was not complicated by BKV reactivation.

A total of 13/25 (52%) BKPyV-positive patients managed with MMF dose reduction suffered from asymptomatic increase in urinary viral loads up to 10^9^ copies/mL, and from BKPyV DNAemia of up to 10^5^ copies/mL, with no decline in renal graft function for the following 1–3 months. All 13 patients were managed with HD-IVIG, as detailed above. Altogether, 12/13 patients (92%) managed with HD-IVIG demonstrated BKPyV clearance within one to five months from HD-IVIG administration, and none showed recurrent BKPyV-viruria/DNAemia or developed BKPyVAN during two to five years of follow-up. HD-IVIG therapy was well tolerated by all patients, with no significant adverse events, except for mild transient headache in six (46%) patients, which responded well to oral paracetamol, and resolved within 24 hours.

One of the 13 patients (8%) showed no decline of BKPyV viral load after two doses of HD-IVIG and required leflunomide treatment.

**Figure 1:**
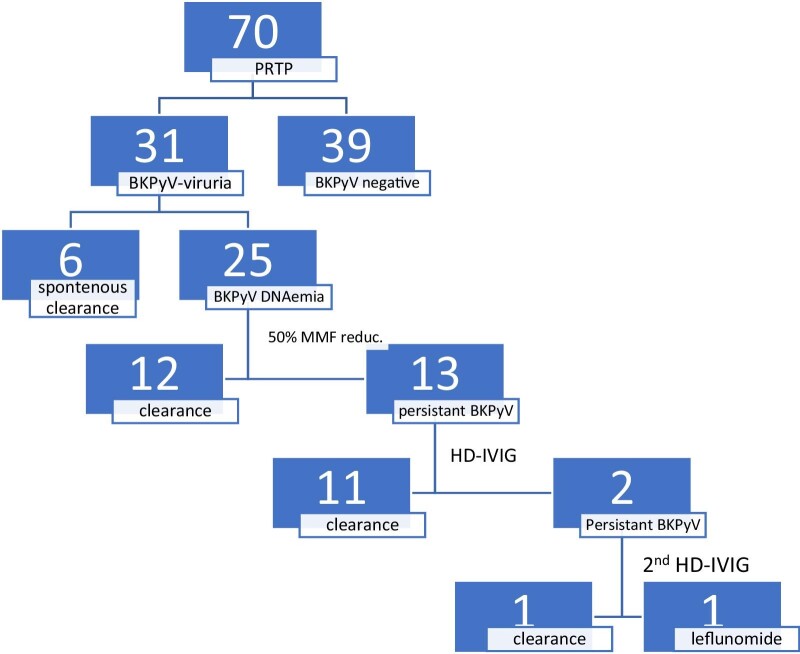
BKPyV trends in pediatric renal transplant patients and treatment strategies.

Of the 13 patients managed with HD-IVIG, two (15%) demonstrated unsatisfactory reduction of BKPyV DNAemia to the level of 1–7.8 × 10^5^ copies/mL. Diagnostic renal biopsies showed no evidence of BKPyVAN and staining for SV-40 was negative. Both patients were managed with a second course of HD-IVIG, given two months apart from the first course. One patient showed complete clearance of BKPyV viruria and DNAemia within several weeks from the second HD-IVIG dose, accompanied by preserved graft function during a five-year follow-up period. The second patient (1/13, 8%) suffered from persistent BKPyV DNAemia >10^4^ copies/mL, albeit an additional dose of HD-IVIG. Given the high risk for BKPyVAN, she was managed with leflunomide (10 mg every other day for 18 months), which was followed by complete resolution of BKPyV DNAemia, and substantial reduction in BKPyV-viruria to 10^4^–10^5^ copies/mL. Despite ongoing BKPyV-viruria, these patients show no recurrent of BKPyV DNAemia, and her graft is well-functioning with no evidence of nephropathy during a three-year follow-up period (Fig. 1).

Data analysis of demographic and clinical characteristics of study participants (Table 1) indicate that BKPyV-positive pediatric renal transplant recipients were of younger median age (10 years compared to 14 years in the BKPyV-negative group), had a slightly higher proportion of female gender (45% compared to 35% in the BKPyV-negative group). Although not statistically significant, girls also were slightly overrepresented in the group of patients requiring HD-IVIG (8/13, 62%). Congenital anomalies of kidney and urinary tract were more prevalent in the BKPyV-positive group (60%, compared to 40% in the BKPyV-negative group). Within the BKPyV-positive group, only one out of the six patients with spontaneous viral resolution received a deceased-donor transplantation. In contrast, the majority of BKPyV-positive patients who required medical intervention (19/25, 76%) were cadaveric-donor recipients. Of the entire cohort, 11/70 patients (16%) required intensified immunosuppression therapy (aka induction with thymoglobulin instead of basiliximab), due immune sensitization prior to transplantation, or acute rejection episodes (treatment with either high-dose steroids or specific anti-rejection treatments). Of these, three patients (27%) were BKPyV-negative, whereas the remaining eight patients (72%) suffered from accelerated BKPyV-replication, necessitating HD-IVIG therapy. All the patients in this cohort were transplanted for the first time.

**Table 1:** Clinical and demographic characteristics of BKPyV-positive and BKPyV-negative pediatric renal transplant recipients.

	All transplanted patients, number (%)	Transplanted patients positive to BKPyV (%)	Transplanted patients negative to BKPyV (%)	
Number of patients	70	31	39	
Median age (years)	13	10	14	*P* = .0134
Female gender	26 (37%)	14 (45%)	12 (30%)	*P* = .215
Congenital anolmalies of urinary tract	30 (42%)	18 (58%)	12 (30%)	*P* = .022
Cadaveric kidney transplant	44 (62%)	23 (76%)	21 (53%)	*P* = .08
Anti-rejection treatment in the past	11 (18%)	8 (26%)	3 (7%)	*P* = .038

## DISCUSSION

To the best of our knowledge, our study is the first to examine clinical and epidemiological characteristics of BKPyV infection in a cohort of pediatric renal transplant recipients in Israel, and to investigate the efficacy of HD-IVIG as a preventative measure for significant BKPyV DNAemia without BKPyVAN.

The overall prevalence ofBKPyV-viruria/DNAemia in our cohort was 47%, with 20% of patients demonstrating accelerated viral replications. These rates are comparable to previous reports. Risk factors for BKPyV-infection in our cohort were also similar to those previously reported, including younger age at the time of transplantation, congenital anomalies of the urinary tract as underlying etiology for native kidney failure, deceased-donor transplantation, and intensified immunosuppressive therapy [4, 5, 21]. Although statistically insignificant, females were overrepresented in the BKPyV-positive group, especially in patients with very high viral loads.

In contrast to the reported 5%–8% prevalence of BKPyVAN in pediatric renal transplant recipients, there was no evidence of BKPyVAN in our cohort of patients, including in high-risk patients with BKPyV DNAemia >10^4^ copies/mL, who are classified as probable or presumptive BKPyVAN [5, 22]. The absence of BKPyVAN may be related either to the relatively small number of patients in our study, to false-negative findings in kidney biopsy, or to the small numbers of kidney biopsies performed in our study. However, we cannot exclude the possibility that the absence of BKPyVAN in our cohort of patients was related to frequent BKPyV viral load monitoring in blood and urine, which led to early detection of patients at risk for accelerated BKPyV replication, and to prompt preventative measures, including MMF dose reduction, followed by HD-IVIG therapy, when indicated. The protocol of BKPyV viral load monitoring used in our study was more stringent than suggested by most guidelines, which recommend less frequent viral load monitored during the first two post-transplantation months, and do not generally recommend routine monitoring of urine BKPyV viral loads [5, 23], given its low specificity for diagnosis of BKPyVAN. Nevertheless, the observed short time frame between kidney transplantation and the appearance BKPyV-viruria in our cohort, combined with the fact that 50% of patients with BKPyV-viruria subsequently develop BKPyV DNAemia, suggests a narrow window of opportunity for prevention of uncontrolled BKPyV replication, which may potentially lead to irreversible graft damage.

While it is widely agreed that immunosuppressive dose reduction is effective in halting BKPyV replication in renal transplant recipients, it harbors a risk of acute graft rejection in 10% of adult and in 15% of pediatric patients undergoing this management [4, 24, 25]. In our study, only one of 31 patients in the BKPyV-positive group (3%) developed acute graft rejection following MMF dose reduction. This low incidence of acute graft rejection may be related to the small number of patients, to the close monitoring of viral load, or to early introduction of preventive HD-IVIG therapy. Moreover, because HD-IVIG is also effective against humoral renal graft rejection, it is not unlikely that this treatment may have potentially prevented asymptomatic early graft rejection in some of our patients with high BKPyV-viral loads who were managed with reduced immune suppression.

The effect of HD-IVIG on BKPyV burden of renal transplant recipients has been previously investigated, generally indicating their efficacy in viral load reduction. However, it is difficult to draw clear conclusions from these studies, due to significant variability in patient characteristics, treatment aims (preventative versus therapeutic), sample size, research methodology, and drug combinations used [17, 26, 27]. Moreover, reports on IVIG therapy in pediatric renal transplant recipients are scarce [18, 19].

Our study has several limitations, including a relatively small number of patients, retrospective methodology, and the absence of a control group, resulting in lack of information regarding the potential proportion of patients who might have developed spontaneous BKPyV resolution, or, alternatively, encountered BKPyVAN or acute graft rejection, without HD-IVIG treatment.

In conclusion, we demonstrate that HD-IVIG therapy is safe and in combination with MMF dose reduction was found to be potentially effective in prevention of irreversible kidney damage in a cohort of pediatric renal transplant recipients with accelerated BKPyV replication. Our findings should be further established by large-scale, prospective controlled trials.

## Data Availability

No new data were generated or analysed in support of this research.
